# Around the Hospitals

**Published:** 1897-09-25

**Authors:** 


### AROUND THE HOSPITALS.

[NOTICE TO THE MANAGERS OF INSTITUTIONS.—Special space is now reserved for the insertion of notices of hospital meetings and festivals. The Editor requests that all notices may reach THE HOSPITAL Office, 28 & 29, Southampton Street, Strand, London, W.C., at least one week before the date of each meeting.]

**Salisbury Infirmary.**—The question of the abuse of the out-patient department has been occupying the attention of the committee during the year ended July 31st last. Two special courts were held during that period to consider the rules for the admission of out-patients, and the result of their deliberations was an expression of the opinion that, though the power of recommending patients was undoubtedly abused at times, it was impossible to lay down a hard and fast rule on the subject, and that the main responsibility must be left to the governors themselves of determining whether those whom they recommended were fitting objects for charity. An outbreak of scarlet fever in the children's ward took place at the end of 1896, caused, it was believed, through the visitors to patients, and since the issue of stringent orders restricting their admission no new cases have occurred. As to the work done during the year, 878 in-patients were admitted, as against 858 admitted during 1895-6; while the number of out-patients has been 1,619, against 1,775; and of casualties 2,368, against 1,683. The daily average number of patients was 91, as against a daily average of 89 in 1895-6; while the daily average number of the household was 40. Financially, the hospital has had a good year, most of the receipts showing a gratifying increase. Subscriptions, however, show a falling-off of over £50 when compared with the previous year, a fact which is all the more alarming when we find that last year they reached the lowest point they have touched since 1888. The hospital did not receive any legacies, but £1,000 in donations was received for the endowment of a cot. The expenditure exceeded the ordinary income by £500, increasing the balance due to the treasurer from £1,400 to £1,900. Without wishing to appear to labour the subject, we would add that here, as at other hospitals which have been reported on under this heading from time to time, the uniform system might with advantage, and with but little difficulty, be adopted in place of, or, if thought desirable, in addition to the accounts at present submitted by the authorities.

**Gloucester General Infirmary.**—The number of patients treated at the Gloucester General Infirmary during 1896 shows a considerable falling off when compared with those for 1895, a matter which is due solely to the epidemic of small-pox at Gloucester in the latter year. In order, therefore, to get some idea of the relative work done at the hospital in 1896 and in previous years it is necessary to leave 1895 out of consideration and compare 1896 with 1894 or 1893. Doing this we find that the number of in-patients admitted in 1893 was 1,409, in 1894 1,374, and in 1896 1,311, a slight decrease, but that the daily average number of beds occupied increased from 83·7 and 83·3 in 1893 and 1894 to 92·7, due to the patients being kept in the hospital for an average of 25·8 days instead of 22·1 and 21·6 in the other years in question. Out-patients numbered 6,939 in 1893, 7,762 in 1894, and 7,048 in 1896. Of the patients admitted in 1896 only 9 per cent. were admitted by letter. The financial condition of the infirmary was fairly satisfactory, the ordinary income being within £50 of the ordinary expenditure, but at this institution also we have to chronicle a falling-off in the annual subscribers. A certain amount of extraordinary expenditure has been incurred, the greater portion of which was due to the fact that the old oak floors of the four wards in the centre block had become worn out and have had to be replaced by polished teak floors. These wards have also been furnished with improved iron bedsteads and cots with hair mattresses. As at Salisbury, the uniform system of accounts might well be adopted.

